# Phylodynamics of HIV-1 from a Phase III AIDS Vaccine Trial in Bangkok, Thailand

**DOI:** 10.1371/journal.pone.0016902

**Published:** 2011-03-10

**Authors:** Marcos Pérez-Losada, David V. Jobes, Faruk Sinangil, Keith A. Crandall, Miguel Arenas, David Posada, Phillip W. Berman

**Affiliations:** 1 CIBIO, Centro de Investigação em Biodiversidade e Recursos Genéticos, Universidade do Porto, Vairão, Portugal; 2 Jobes Consulting Service, Rockville, Maryland, United States of America; 3 Global Solutions for Infectious Diseases, South San Francisco, California, United States of America; 4 Department of Biology, Brigham Young University, Provo, Utah, United States of America; 5 Departamento de Bioquímica, Genética e Inmunología, Universidad de Vigo, Vigo, Spain; 6 Department of Biomolecular Engineering, University of California Santa Cruz, Santa Cruz, California, United States of America; University of Nebraska, United States of America

## Abstract

**Background:**

In 2003, a phase III placebo-controlled trial (VAX003) was completed in Bangkok, Thailand. Of the 2,546 individuals enrolled in the trial based on high risk for infection through injection drug use (IDU), we obtained clinical samples and HIV-1 sequence data (envelope glycoprotein gene gp120) from 215 individuals who became infected during the trial. Here, we used these data in combination with other publicly available gp120 sequences to perform a molecular surveillance and phylodynamic analysis of HIV-1 in Thailand.

**Methodology and Findings:**

Phylogenetic and population genetic estimators were used to assess HIV-1 gp120 diversity as a function of vaccination treatment, viral load (VL) and CD4^+^ counts, to indentify transmission clusters and to investigate the timescale and demographics of HIV-1 in Thailand. Three HIV-1 subtypes were identified: CRF01_AE (85% of the infections), subtype B (13%) and CRF15_AE (2%). The Bangkok IDU cohort showed more gp120 diversity than other Asian IDU cohorts and similar diversity to that observed in sexually infected individuals. Moreover, significant differences (*P*<0.02) in genetic diversity were observed in CRF01_AE IDU with different VL and CD4^+^ counts. No phylogenetic structure was detected regarding any of the epidemiological and clinical factors tested, although high proportions (35% to 50%) of early infections fell into clusters, which suggests that transmission chains associated with acute infection play a key role on HIV-1 spread among IDU. CRF01_AE was estimated to have emerged in Thailand in 1984.5 (1983–1986), 3–6 years before the first recognition of symptomatic patients (1989). The relative genetic diversity of the HIV-1 population has remained high despite decreasing prevalence rates since the mid 1990s.

**Conclusions:**

Our study and recent epidemiological reports indicate that HIV-1 is still a major threat in Thailand and suggest that HIV awareness and prevention needs to be strengthened to avoid AIDS resurgence.

## Introduction

HIV/AIDS emerged late in Thailand compared to other countries worldwide [1]. The first case was reported in 1984, although this was a returned emigrant who developed AIDS elsewhere [2]. A few more cases were reported in 1984–1988 between men who had sex with men (MSM) and injecting drug users (IDU) [3]. In 1989 AIDS hit Thailand hard after HIV spread very quickly through the IDU community, and a year later entered the commercial sex worker (CSW) networks [1], [4]. In subsequent years, prevalence rates among these high-risk groups grew explosively from almost zero to 30 to 50% [5]–[7]. Since then, the Thai HIV epidemic has been largely driven by CSW and IDU [1], whose epidemics appear to be linked [8], [9]. Over the last 12 years, for example, heterosexual (HT) and IDU transmissions accounted for 80–85% and 5% of the infections, respectively [3]–[5]; and although the former has now diminished considerably, the latter has remained high [7].

In 1991, AIDS prevention became a national priority in Thailand and between 1993 and 1997 the government increased the national budget, launched several campaigns to control and inform about AIDS spread (Ministry of Public Health, Thailand; eng.moph.go.th) and initiated the “100 percent condom program” [10]. All these policies slowed down the spread of AIDS and the national prevalence rate was reduced by 0.6% points [7]. These AIDS campaigns were mostly successful at reducing HIV infections in CSW, whose prevalence rate is now only 5%, but older HIV epidemics in IDU and MSM continue unabated (prevalence rates are >25%; [7]), fueling epidemics in CSW [11] and causing new outbreaks [1]. Almost 1.5% adults are still infected with HIV in Thailand (∼610,000 infected individuals) making AIDS a leading cause of death (30,000 deaths in 2007; [6], [7]). The Thai HIV epidemic has become now more heterogeneous [12] and it is increasingly affecting people traditionally considered to be at lower risk of infection [7]. Of even more concern, there are already signs that the epidemic could grow in coming years: prevalence rates among high-risk groups have increased, condom use has decreased, and risky sexual behavior is on the rise [7], [13].

In the early years of the AIDS epidemic in Thailand HIV-1, subtypes were segregated by risk group. Subtype B was predominant in IDU; while CRF01_AE (a recombinant between subtypes A and E) was predominant in HT and MSM [9], [14]. As the Thai epidemic progressed, CRF01_AE increased in frequency across all high-risk groups [15], [16] and by 1995 it became also the predominant subtype in IDU [17]. Thus, between 1995 and 2004 CRF01_AE accounted for 80–97% (depending on the study) of the new HIV-1 infections [3], [18]. But the Thai molecular epidemiology has been gradually growing in complexity and now it seems to be entering a new phase [3], [9]. New recombinant CRF15_01B, CRF01_AE/B and CRF01_AE/C isolates are constantly being identified both within HT and IDU [3], [9], [19]–[22] and some may be increasing their frequency rapidly (13% CRF01_AE/B in IDU; [9]). Under this new epidemic scenario, molecular surveillance becomes crucial to monitor emerging trends in HIV transmission, assess intervention strategies, and evaluate vaccine efficiency and design [9], [22]–[25].

HIV spreads through often complex contact networks or transmission (infection) chains [26], [27]. The characteristics of such networks play a crucial role in determining short- and long-term disease dynamics [28]; hence, understanding those networks may translate into more efficient prevention measures and treatment interventions [29], [30]. Several phylogenetic studies suggest that transmission chains associated with acute (early) HIV-1 infection may greatly contribute to viral transmission and spread of the epidemic [31]. Data from both sexually- and drug-related acute infections in Europe [31]–[36], Canada [37], [38], and Panama [39] have reported clustering in 24–65% of HIV-1 sequences. However, in a genetic analysis of 130 early diagnosed HIV-1 infections in IDU from Bangkok only 7.4% of the subtype B and 16.5% of the CRF01_AE isolates formed transmission clusters [40]. Similarly, in a recent study of sexually infected HIV-1 patients (mostly MSM) in North America, clustering was detected in only 11% of the isolates [41]. Therefore, it seems like the extent to which acute transmission of HIV-1 is clustered remains open.

Thailand is one of the key international partners in the HIV vaccine efficiency trials with three trials already completed [42]–[44]. In 2003, the first phase III placebo-controlled trial (VAX003) of a candidate HIV-1 vaccine (AIDSVAX B/E) was completed in individuals at high risk for HIV-1 infection [45]–[47]. The study enrolled 2,546 uninfected IDU from and around Bangkok of which we obtained clinical samples for 215 who became infected with HIV-1 between 1999 and 2003 despite intensive risk reduction counseling. Plasma samples from these individuals were obtained within the first 13 months after infection, and envelope glycoprotein (gp120) viral sequences were generated. These sequences (the “VAX003 dataset”) have recently been released to the scientific community through the Global Solutions for Infectious Diseases HIV sequence Database (www.GSID.org).

Here, we analyze the VAX003 data to assess HIV-1 variation as a function of treatment (vaccine or placebo), viral load, and CD4^+^ counts. Moreover, we perform a molecular surveillance of the VAX003 gp120 dataset to identify HIV-1 circulating subtypes in Bangkok and infer transmission networks in IDU. Finally, we combine the VAX003 dataset with other Thai sequences available in the HIV Los Alamos database (www.hiv.lanl.gov) to investigate the timescale and molecular population dynamics of HIV-1 in Thailand. The VAX003 dataset is the largest collection of gp120 sequences from infections resulting from new and recent transmissions in Thailand and one of the few datasets collected from a large IDU cohort. These data provide a unique opportunity to study HIV-1 evolution in an epidemiological context and we anticipate it will contribute to the analysis and interpretation of the results from the RV144 Phase III HIV vaccine trial recently completed in Thailand [48], [49].

## Results

### Molecular surveillance and subtype diversity

We indentified 182 CRF01_AE (84.7%), 29 subtype B (13.4%), and 4 discordant isolates that presumptively are CRF15_AE (1.9%). This latter recombinant type is mostly CRF01_AE but also includes most of gp120 (except for approximately the first 36 nucleotides) and the external portion of gp41 from subtype B [8]. Full genome sequencing of these discordant HIV isolates are needed to confirm this result. Number of isolates (as percentages) per year (1999 to 2003) was similar within each subtype (Table S1).

Estimates of genetic diversity (θ) were similar (∼0.11) across subtypes (Table 1). Selection estimates (ω) were generally below 1 although subtype B [ω_PAML_ = 0.777; ω_omegaMap_ = 0.778 (0.673–0.901)] showed higher (and significant for omegaMap) average *d*_N_/*d*_S_ rates than CRF01_AE [ω_PAML_ = 0.580; ω_omegaMap_ = 0.404 (0.366–0.443)]. Population recombination rates (ρ_omegaMap_), however, were significantly lower for subtype B [3.95 (3.45–4.53)] than for CRF01_AE [15.56 (14.65–16.65)]. DNA genetic divergence (±SD) was higher for subtype B (0.096±0.019) than for CRF01_AE (0.067±0.011). θ, ω_PAML_ and genetic divergence were also estimated in the North American VAX004 gp120 dataset [50] for comparison between B subtypes (Table 1). θ estimates were again similar between datasets, but ω_PAML_ estimates were lower for the VAX004 dataset (0.432) than for the VAX003 dataset (0.777), while genetic divergence was significantly higher for the VAX004 dataset (0.112±0.015).

**Table 1:** Overall subtype diversity estimates.

HIV-1	θ	ω_PAML_	ω_omegaMap_	ρ_omegaMap_	GD
VAX003-CRF01_AE (182)	0.110	0.581	0.404	15.56	0.067
			(0.366–0.443)	(14.65–16.65)	(0.067–0.067)
VAX003-Subtype B (29)	0.112	0.777	0.778	3.95	0.096
			(0.673–0.901)	(3.45–4.53)	(0.094–0.098)
VAX004-Subtype B (345)	0.105	0.432	-	-	0.112
					(0.112–0.112)

Genetic diversity (θ), selection in PAML (ω_PAML_) and omegaMap (ω_omegaMap_), population recombination rate in omegaMap (ρ_omegaMap_), and genetic divergence (GD). Number of isolates analyzed is indicated between parentheses in the first column. Estimates from the North American VAX004 subtype B trial were included for comparison.

### Phylogenetic analysis

**Figure 1:**
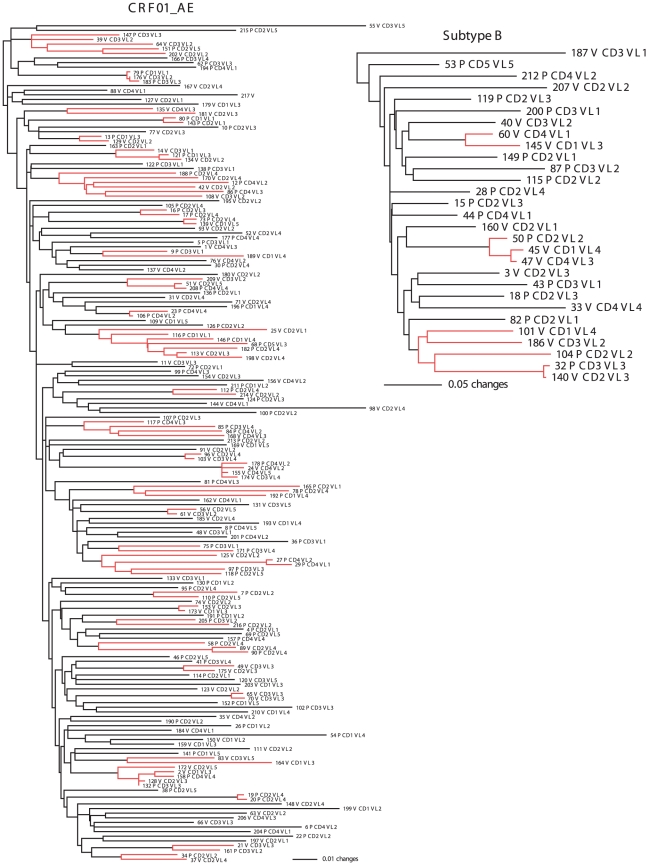
HIV-1 subtype phylogenetic trees. Maximum likelihood phylogenetic inference of Bangkok HIV-1 CRF01_AE and subtype B population structuring as a function of treatment [placebo (P) and vaccine (V)], viral load (VL), and CD4^+^ counts. Branch lengths are shown proportional to the amount of change along the branches. Clades supported by bootstrap proportions ≥70% and posterior probabilities ≥0.95 in the Bayesian analysis (transmission chains) are shown in red color and their terminals in bold. Only one clone per isolate (numbered) is represented for simplicity.

The GTR+Γ+I model [51] was chosen as the best-fit model for both the VAX003 gp120 dataset and for all their corresponding codon-position partitions. ML and Bayesian phylogenies did not show any obvious structure based on treatment, VL or CD4^+^ categories (Fig. 1). Individuals within each factor seemed to be randomly distributed across the phylogeny.

### Transmission clusters

ML and Bayesian phylogenetic analyses of HIV-1 subtype B and CRF01_AE showed 3 and 31 well-supported clades (bootstrap proportions ≥70% and posterior probability ≥0.95), respectively (Fig. 1). These transmission networks involved 10 (34.4%) subtype B and 91 (50%) CRF01_AE IUD isolates distributed in 2 small (<5 isolates)/1 large (≥5 isolates) and 26/5 clusters, respectively (Table S2). Attendance to a particular clinic and estimated date of seroconversion (considered as a time window of ≤6 months) were found to be associated with 1 and 1 subtype B clusters, respectively, and 6 and 13 CRF01_AE clusters, respectively. Some overlap between factors was observed in some clusters (e.g., clade 2 in subtype B and clade 7 in CRF01_AE). Nonetheless, these results suggest that between 1999 and 2003, the estimated date of infection seemed to play a larger role than geographic location at establishing transmission chains in CRF01_AE IDU from Bangkok (Table S2).

### Viral evolution and patient factors

Average θ, ρ, and ω intra-patient estimates within categories were very low for both subtypes (Table 2). For most CRF01_AE datasets ω>1, while for the rest ω≈1. On the contrary, for most subtype B datasets ω<1, but ω>1 was also found in several cases. These intersubtype differences, nonetheless, were non-significant. CRF01_AE sequences from individuals with lower VL and higher CD4^+^ counts showed lower θ values (0.005–0.006) than individuals with higher VL (*P* = 0.016) and lower CD4^+^ (*P* = 0.007) counts (0.007–0.009). These two factors were inversely correlated (Pearson correlation coefficient = −0.218, *P* = 0.003).

**Table 2:** Mean patient diversity estimates.

HIV-1	θ	ρ_LDhat_	ω_PAML_	ω_HYPHY_
Subtype CRF01_AE				
Treatment				
Placebo (92)	0.007	4.8	1.045	1.208
Vaccine (87)	0.007	3.0	1.057	1.229
VL Categories (RNA copies/mL)				
1: <1×10^4^ (29)	0.005	6.1	0.999	1.150
2: 1×10^4^–5×10^4^ (48)	0.006	3.7	1.030	1.198
3: 5×10^4^–10×10^4^ (40)	0.007	2.8	0.958	1.110
4: 10×10^4^–25×10^4^ (45)	0.008	4.4	1.265	1.451
5: >25×10^4^ (21)	0.009	2.5	0.905	1.082
CD4^+^ counts (cells/mm^3^)				
1: <3×10^2^ (31)	0.009	2.4	0.921	1.082
2: 3×10^2^–5×10^2^ (79)	0.007	3.8	1.100	1.250
3: 5×10^2^–7×10^2^ (39)	0.006	3.7	1.003	1.187
4: >7×10^2^ (34)	0.006	5.9	1.119	1.317
Subtype B				
Treatment				
Placebo (17)	0.008	5.5	0.775	0.869
Vaccine (14)	0.008	4.4	0.978	0.997
VL Categories (virions/mL)				
1: <1×10^4^ (8)	0.004	3.8	0.653	0.707
2: 1×10^4^–5×10^4^ (9)	0.009	2.3	0.873	0.887
3: 5×10^4^–10×10^4^ (7)	0.011	11.4	0.998	1.199
4: >10×10^4^ (7)	0.007	1.9	1.100	1.047
CD4^+^ counts (cells/mL)				
1: <3×10^2^ (31)	0.011	12.4	0.870	0.899
2: 3×10^2^–5×10^2^ (79)	0.006	5.2	0.847	0.950
3: 5×10^2^–7×10^2^ (39)	0.010	2.0	0.762	0.818
4: >7×10^2^ (34)	0.006	4.3	1.121	1.078

Genetic diversity (θ), population recombination rate in LDhat (ρ_LDhat_), and selection estimates in PAML (ω_PAML_) and HYPHY (ω_HYPHY_). Number of isolates analyzed is indicated between parentheses.

### Population dynamics

**Figure 2:**
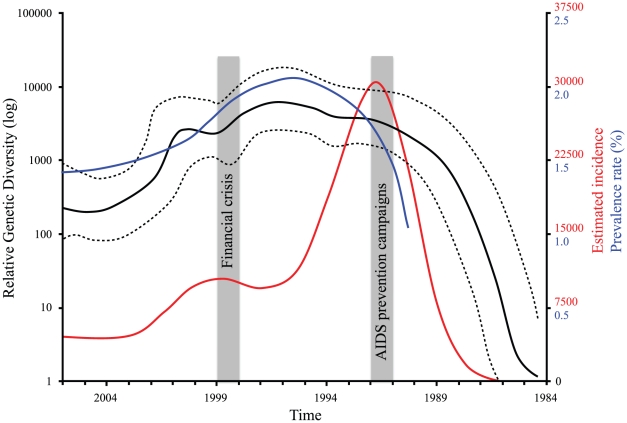
HIV-1 CRF01_AE past population dynamics. Bayesian skyline plot of the HIV-1 CRF01_AE subtype in Thailand. Solid black lines show the median estimate and dashed black lines the 95% high posterior density limits. The estimated incidence and prevalence rate are indicated in red and blue, respectively (see text for details).

BEAST's estimate of the substitution rate was 0.0055 (0.0050–0.0060) for CRF01_AE and 0.0027 (0.0015–0.0038) for subtype B. The Most Recent Common Ancestor (MRCA) was dated in 1984.5 (1983–1986) for CRF01_AE and in 1965 (1950–1979) for subtype B. The BSP analysis of CRF01_AE sequences (Fig. 2) suggested that the relative genetic diversity increased exponentially between 1984 and 1991, moderately between 1992 and 1995, decreased between 1996 and 2004 with a spike in 1999–2000, and then increased slightly between 2005 and 2006 (the age of our most recent sample).

## Discussion

### Molecular surveillance and subtype diversity

The predominant HIV-1 subtype circulating in IDU (215 patients) from Bangkok during 1999–2003 was CRF01_AE (85%). Subtype B accounted for 13% of the infections and CRF15_AE for 2%. Two early (1995 to 1998) molecular surveys in Bangkok [17], [40] including 102 and 130 IDU, respectively, and using the C2-V4 *env* region (345 bp), reported also high percentages of subtype B isolates (20–21%) but no CRF15_AE recombinants. Additional surveillances among IDU in Bangkok [21] during 1997–1998 (111 patients) using *env* (530 bp) and protease (297 bp) still detected high percentages of subtype B isolates (23%), but also 3.6% CRF15_AE isolates. Interestingly in Northern Thailand, a near full HIV-1 genome study (1999–2002; 38 patients) among IDU detected an increasing proportion of CRF15_AE (13% total) infections but no pure subtype B isolates, suggesting that the latter subtype became extinct in this region [9]. Although full HIV-1 genome surveys increase the probability of finding intersubtype recombinants, our surveillance from 1999 to 2003 suggests that subtype B is declining and CRF15_AE is increasing among IDU from Bangkok as previously predicted by others [24] and observed in other high-risk groups across the country [3], [22], [52]. Nonetheless, considering subtype B prevalence rate and genetic diversity (Table 1), it may remain circulating in Thailand for many years. This information is important to ensure that the virus diversity upon which vaccines are designed matches the circulating viral population. Fortunately, vaccine candidates used in the RV144 Phase III HIV vaccine trial largely contain both subtype B and CRF01_AE viruses [48], [49].

Our estimate of gp120 genetic divergence in CRF01_AE viruses (0.067±0.011) from IDU was higher than previously reported for *env* (0.059±0.011) in Northern Thai IDU patients [9], but much lower than those reported for the C2-V4 *env* region (mean: 0.109–0.150) in other Thai regions among mostly (95%) sexually infected individuals [3]. These comparisons must be considered with caution since the *env* regions compared are not exactly the same and we removed many of the variable sites after the GBlocks analysis. Genetic divergence estimates using the full VAX003 gp120 alignment were of 0.100±0.015. But independently of what dataset we consider, both Tovanabutra et al. [9] and our own estimates are higher than those reported in other Asian IDU groups in, for example, China [53] or Vietnam [54], [55], and closer to those observed in sexual transmission cohorts [3], where diversity is generally higher [56]. This result then suggests that the IDU epidemic in Thailand is likely to be mature and that extensive exchange between sexual and IDU exposures and transmissions has been ongoing for years [9], which is also supported by our phylogenetic results below.

gp120 subtype B sequences from Bangkok are significantly less divergent than those from the North American VAX004 vaccine trial (Table 1). This and previous gp120 CRF01_AE estimates indicate that Thai HIV-1 populations are more homogeneous than those observed in other areas like Vietnam (see below) or North America. The increased homogeneity of viruses in Bangkok has been attributed to the relatively recent introduction of HIV in Thailand (1984) and a pronounced founder effect resulting from the rapid spread of the virus [1], [4]. This result then suggests a greater opportunity to overcome the challenge of HIV diversity [57] and to detect protective immunity induced by candidate vaccines in Thailand compared to North America or Africa, where viral genetic diversity is much higher. Indeed, the outcome of the RV144 vaccine testing in Thailand seems to have had greater success by better coverage of this limited diversity with vaccinated volunteers showing 31.2% fewer infections than placebo recipients [48], [49]. American subtype B viruses also appear to be under stronger purifying selection (ω = 0.43–0.58) than the Thai subtype B viruses (0.78). This suggests that differences could exist in the intrinsic immune response among ethnicities [58] or transmission type (i.e., IDU vs MSM) [59].

Our genetic estimators indicate that CRF01_AE experienced almost four times more recombination than subtype B (Table 1). Consequently, one could also expect that higher recombination rates would inflate ω_PAML_ estimates [60]–[62], but that does not seem to be the case, since subtype B showed significantly higher levels of selection than CRF01_AE for all estimators. Similarly, CRF01_AE presented a mean substitution rate per site twice as high as that observed for subtype B. Significant differences in adaptive selection and substitution rate between HIV-1 subtypes have been reported before [59], [63] and were attributed to differences in immune selective pressure from the host and in mutation rate or generation time of the virus.

### Phylogenetic structure of HIV-1 in Thailand

Our CRF01_AE and subtype B phylogenetic trees suggest that HIV populations in IDU from Bangkok are not structured by any of the epidemiological and clinical factors studied (Fig. 1). Moreover, our BEAST analyses of both VAX003-LA gp120 subtypes did not show phylogenetic structuring based on transmission type either (Fig. S1). These results agree with previous CRF01_AE star-like phylogenies reported in IDU from Bangkok [40]. Geographically broader CRF01_AE phylogenetic studies in Central [64], Northern [9] and across Thailand [3], [22] also showed lack of structuring based on transmission type, sociodemographic factors and geographic location. In the Wirachsilp et al. [3] study, for example, sequences from Bangkok clustered together with sequences from other regions. Similarly, Keele et al. [65] also showed that viral *env* genes evolving from individual transmitted or founder HIV-1 subtype B viruses generally exhibited a star-like phylogeny, such as the one observed in North American viruses [41].

Given the age of the HIV-1 epidemic in Thailand and the fact that the virus is thought to mutate at a rate of 1% per year [66], [67], the possibility existed that different clades would have emerged in different regions or high-risk groups in Thailand. Indeed phylogenetic structuring based on these factors has been observed before between subtypes in, for example, Africa [68] and Asia [69] and within subtypes in, for example, Vietnam [54] and China [70]. But contrary to what happened in those HIV/AIDS epidemics, the Thai epidemic spread exponentially across the whole country and risk types [1], which could erase early genetic differentiation and results in star-like gene genealogies [71], [72]. Moreover, both molecular (this study and [8], [9]) and Thai behavioral [11], [73] data indicate that bridging between drug and sexual epidemics through CSW has been ongoing for years, which again reduces the opportunity for differentiation.

### Phylogenetic clusters in acute transmissions

The extent to which acute transmission of HIV-1 is clustered is not clear. Some studies [31]–[38], [74] report high clustering (24 to 65%) levels, while others [40], [41] show much lower values (7 to 17%) for the same subtypes and transmission routes. Our more comprehensive phylogenetic analyses of IDU from Bangkok show higher proportions of early subtype B (35%) and CRF01_AE (50%) infections falling into clusters, confirming that transmission chains associated with acute infection play a key role in HIV-1 transmission and spread [31]. Transmission clusters in Nguyen et al. [40] were inferred using the C2-V4 *env* region (345 bp). This gene region, although broadly used in HIV genetic studies, is less informative than the gp120 (∼1.5 kb) region used here for estimating phylogenetic clustering. As for Pérez-Losada et al. [41], that study covered North America, while our Thai study and others before, focus on a single city, a small country or a recently infected area. This suggests that the size and population structure of the studied area affect our ability to identify HIV-1 transmission chains. Moreover, differences in clustering have been also observed between subtypes, transmission routes and regions [33], [34], [41]. Hence future HIV vaccine trials should pay attention to potential sources of clustering that can effectively render samples non-independent.

### Viral evolution and patient factors

No significant differences in recombination, mutation, and selection rates were observed among vaccinated and placebo individuals in both subtype B and CRF01_AE. This is consistent with the overall outcome of the VAX003 trial where immunization with AIDSVAX B/E did not significantly affect the rate of infection, the VL, the CD4^+^ count, or the clinical outcome of vaccine recipients compared to placebo recipients [45]. Lower VL and higher CD4^+^ counts, however, were significantly associated with lower mutation rates in CRF01_AE (Table 2). Since genetic diversity may be positively correlated with N_e_, one could expect that greater VL (census size) would also cause an increase on the number of effective virions [41].

### Population dynamics of Thai HIV-1 subtypes

Previous full-genome phylogenetic analyses of HIV-1 CRF01_AE in Southeast Asia [54] indicate that CRF01_AE was introduced from Africa to Thailand and then spread elsewhere. Our coalescent estimate of the time of emergence of CRF01_AE in Thailand was 1984.5 (1983–1986). An slightly earlier estimate (1981±2 years) was previously reported by Liao et al. [54] using the same method but including 64 near full-length CRF01_AE nucleotide sequences from Africa, China, and Vietnam. Hence, both studies suggest that CRF01_AE was circulating cryptically in Thailand for 3–10 years before it was first detected in 1989 [75]. Similar time lags between evolutionary estimates and the recognition of symptomatic patients have been observed before in other countries such as United States [76] and Vietnam [54]. In Western countries, the estimated median incubation period before AIDS development in the absence of antiretroviral therapy is 10–12 years [77], although in Thailand a shorter incubation period (7 years) has been suggested [78]. HIV testing in Thailand started in 1985 and only 3 cases were detected [79]. There were no cases reported in 1986, but many thousands were detected over the next 3 years, particularly among IDU from Bangkok [75]. By February 1990, almost 15,000 cases of HIV-1 infection have been already documented across the country [80]. Similarly to what happened in other regions, CRF01_AE could have been introduced in Thailand years before its detection in 1989.

Phylogenetic analyses of HIV-1 subtype B *env* data collected worldwide [76] indicate that this subtype was introduced from Africa to Haiti and then spread elsewhere (pandemic clade). In that study, the Thai subtype B isolates did not seem to form a separate cluster (independent HIV-1 expansion), hence our coalescent estimate of the time of emergence of subtype B (1965±15) approximates that of the emergence of the subtype worldwide (1968–1969±3 years) [41], [76]. Discrepancies between these and our current estimate are probably due to differences in sample size: the subtype B dataset analyzed here is geographically more restricted and includes fewer sampling points. A short interval of sampling years, for example, provides less information about the average rate during that interval than does a long interval [63], [81]. The larger HPD intervals of the Thai estimate supports that idea.

Our BEAST analysis of CRF01_AE past dynamics (Fig. 2) agrees well with the history of HIV/AIDS spread in Thailand and the prevalence and incidence rates reported [6] and predicted using backcalculation models [82]. Prior to 1987 the prevalence of HIV in Thailand was low, but once HIV entered the MSM, IDU and CSW networks (1988–1993) prevalence rates exploded rising from virtually zero to up to 50% [4]–[7] and so did the relative genetic diversity (N_e_τ). In 1991, AIDS prevention became a national priority at the highest level and several campaigns were launched to control AIDS spread (Ministry of Public Health, Thailand; http://eng.moph.go.th/). Consequently, prevalence rates began to decline soon after (Fig. 2; see also [10]) and HIV incidence was reduced by a third [82]. Concomitantly, N_e_τ leveled off and slowly began to decline in 1996. In 1998, due to the Asian Financial Crisis, HIV/AIDS funding was severely reduced [83] and many programs like the HIV prevention schemes were downscaled or suspended [7], [83], [84]. This led to a decline in awareness and possibly an increase in unsafe sexual behavior [7]. Consequently, the incidence rate spiked for two years and so did N_e_τ. In 2002 Thailand launched the third National Plan for the Prevention and Alleviation of HIV/AIDS (Ministry of Public Health, Thailand; eng.moph.go.th). Consequently, both incidence and N_e_τ decreased again until 2004, but since then the former has remained constant and the latter has increased slightly and remained relatively high. Under circumstances of low surveillance and high HIV diversity, new or existing infective strains could expand exponentially and provoke a resurgence of AIDS across the country. There are already signs that the epidemic could grow in coming years [7], [13]. More importantly, the epidemic has never eased off among certain groups like IDU, where infection rates are still very high (∼30%; [7]) and continue to be a reservoir for HIV fueling old and causing new epidemics [1], [11]. Thailand must then increase prevention efforts, especially among high-risk groups such as IDU and MSM, but also among the general population since the AIDS epidemic seems to be more heterogeneous now [12]. In light of these concerns, the current government has increased HIV/AIDS prevention efforts. In 2007, a three-year strategic plan was announced that would focus on those most at risk of HIV infection and difficult-to-reach groups [85]. How these new policies are going to affect HIV-1 diversity and dynamics is for further studies to see.

## Materials and Methods

### VAX003 vaccine trial participants

The 2,546 volunteers participating in the VAX003 trial (NCT00006327) were recruited from 17 clinics in and around Bangkok [45]–[47]. They all were considered at high risk for HIV-1 infection through injection drug use. The vaccine trial protocol did not specify racial categories and no effort was made to distinguish linguistic and geographic groups. Volunteers were randomly assigned to vaccine or placebo groups according to a 1∶1 ratio. All subjects were immunized with AIDSVAX B/E, a bivalent vaccine prepared by combining purified recombinant gp120s from two different strains of the HIV-1 virus incorporated in alum (aluminum hydroxide) adjuvant: the subtype B strain (MN) and the subtype CRF01_AE isolate (A244). All subjects were immunized according to a 0, 1, 6, 12, 18, 24, and 36-month schedule. Serum samples were collected immediately prior to each injection and two weeks after each injection, with a final blood sample taken at 6 months following the final injection. The specimen taken prior to each injection was used to calculate pre-boost anti-gp120 titer values and submitted for HIV testing (ELISA). The immunoassays selected for HIV diagnosis were unaffected by antibodies to the AIDSVAX B/E antigens. If evidence of HIV infection was obtained, confirmatory testing was carried out by immunoblot. Once HIV-1 infections were confirmed, HIV-1+ subjects were enrolled in a separate protocol (Step B) where plasma and cells were collected at regular intervals for up to two years post infection. Plasma samples were used for measurement of viral loads and envelope glycoprotein sequencing. Frozen lymphocytes were cryopreserved for immunologic and genetic testing. The date of infection was defined as the midpoint between the last seronegative specimen and the first seropositive specimen. The estimated time of infection ranged from 0 to 13 months with a mean time of infection of 3–4 months. Viral load (VL) and CD4^+^ measurements were taken and patients were subdivided into 4 or 5 categories for genetic analyses (see Table 2).

### Molecular datasets

Of the 2,546 volunteers enrolled in the trial 230 became infected with HIV-1 [45] and we obtained clinical samples for 215 of them. Three to six clones per individual were collected from the same earliest post-infection plasma sample and sequenced for the viral gp120 gene (665 sequences total). A listing of the sequence data used for this analysis has recently been released online and can be accessed at www.gsid.org. All gp120 sequences were determined using an ABI 3100 sequencer and assembled using Sequencher (www.genecodes.com).

HIV-1 subtype was determined using the REGA HIV Subtyping Tool 2.0 [86] and the Recombinant Identification Program: RIP 3.0 [87] at Los Alamos (http://hiv-web.lanl.gov/content/index). Discordant (intersubtype recombinants) isolates were visually inspected and confirmed in RDP 3.0 [88], [89]. Two main HIV-1 subtypes were identified, CRF01_AE (182 isolates) and subtype B (29 isolates) (see the Molecular Surveillance and Subtype Diversity section). Because of their genetic and epidemiological differences [90], these subtypes were analyzed separately. For population dynamic analyses full VAX003 gp120 sequences (only one clone per patient) were combined with other full length, dated Thai gp120 sequences from the Los Alamos database as of January 2010 to generate final datasets of 343 CRF01_AE (from 1990 to 2006) and 47 subtype B (from 1990 to 2003) isolates. These combined datasets included 217/34 IDU, 36/4 HT, 3/0 CSW and 87/9 unknown risk-group CRF01_AE/subtype B isolates.

### Sequence alignment

Nucleotide sequences were translated into amino acids and aligned in MAFFT 5.7 [91] using the global algorithm (G-INS-i). Ambiguous regions in the resulting alignment were identified and removed using GBlocks 0.91b [92]. Conserved amino acid regions were translated back to nucleotides generating alignments of 1,317–1,329 sites for CRF01_AE and 1,398–1,413 sites for subtype B. Full gp120 sequences (1,497–1,629 bp) were analyzed for each patient, in which case the alignments were trivial.

### Phylogenetic analysis

The best-fit model of DNA substitution was selected with the Akaike Information Criterion [93] as implemented in jModelTest 1.0 [94]. Maximum likelihood phylogenetic trees were inferred in RAxML 7.0.3 using 3 codon-position partitions [95]. Nodal support was assessed using the bootstrap procedure [96] with 1,000 replicates. Heuristic searches were performed under the best-fit model. In addition, Bayesian trees were inferred in MrBayes 3.1.1 [97] using also 3 codon-position partitions. We ran four chains (one cold and three heated) for 2×10^7^ generations, sampling every 1,000 steps. Each run was repeated twice. Convergence and mixing of the Markov chains were assessed in Tracer 1.5 [98].

Phylogenetic transmission (infection) clusters [29] were defined as those clades with bootstrap proportions ≥70% and posterior probabilities ≥0.95. Attendance at a particular clinic (which served as proxy for location of residence) and estimated date of seroconversion were screened for all the isolates contributing to clusters. Genetic divergence estimated as the mean pairwise genetic distances under the K2P model [99] was also calculated for comparison with previously published estimates.

### Genetic estimates and patient factors

The VAX003 trial included vaccinated and non-vaccinated individuals with different VL and CD4^+^ counts. These individuals do not constitute natural populations, therefore, all genetic estimators described in this section were either applied to intra-patient datasets (3 to 6 clones) or full-subtype datasets (29 subtype B and 182 CRF01_AE isolates). Genetic diversity (θ) and population recombination rate (ρ) was estimated for each patient using LDhat 2.1 [100]. Here, each analysis was repeated 10 times and the ρ mean estimate was used for subsequent analyses. Molecular adaptation was assessed using the ratio of nonsynonymous (*d*_N_) to synonymous (*d*_S_) substitution rates (ω) and estimated using the model M0 (one-ratio) in PAML 3.14 [101] and Fixed Effects Likelihood (FEL) in HYPHY 1.0 [102]. In the latter case, recombination was taken into account by first detecting recombination breakpoints with GARD [103] and then estimating the *d*_N_/*d*_S_ ratios independently for each fragment. Simultaneous estimation of ω and ρ was also performed in omegaMap [62] for the full-subtype datasets.

Average estimates of ρ, θ, and ω were compared across factors (e.g., vaccinated and placebo; see Table 2) using the Kruskal-Wallis test in Aabel 3 (www.gigawiz.com). Tests based on linear models (e.g., ANOVA) were not applied because their underlying assumptions were not met by some of the data sets.

### Population dynamics

Past population dynamics of CRF01_AE in Thailand was inferred in BEAST 1.5.3 [104] using the Bayesian Skyline Plot (BSP) model [105] and a relaxed clock (lognormal) model of rate of substitution [106]. BSP searches showed overdispersed 95% High Posterior Density (HPD) intervals for subtype B, hence the exponential growth model was used instead. Relative genetic diversity through time (N_e_τ) was estimated directly from dated isolates under the best-fit model of nucleotide substitution. The hyperparameter *m* (number of grouped intervals) was set up 1/4 of the sequences in each case. Two runs 10^8^ and 2×10^7^ generations long were completed for each CRF01_AE and subtype B, respectively. All output generated by BEAST was analyzed in Tracer 1.5 to test for convergence and mixing and implement the BSP model.

## Supporting Information

Figure S1 **HIV-1 BEAST Bayesian trees.** BEAST maximum clade credibility trees of Thai HIV-1 CRF01_AE (large tree) and subtype B (small tree) isolates. Injecting drug users (red), heterosexuals (blue), commercial sex workers (green), and unknown risk group (black) infections are indicated. Branch lengths are shown proportional to the amount of change along the branches. Only one clone per patient is represented for simplicity.
(EPS)

Table S1 **Number of isolates/percentage per year and subtype.**
(DOCX)

Table S2 **Phylogenetic transmission clusters.** Estimated date of infection and clinical site for subtype B and CRF01_AE.
(DOCX)
