# Supplementary material for: Phylodynamics of HIV-1 from a Phase III AIDS Vaccine Trial in Bangkok, Thailand
Source: PLoS One. 2011 Mar 10;6(3):e16902. doi: 10.1371/journal.pone.0016902 (PMC3053363; doi:10.1371/journal.pone.0016902)
Supplement: Table S1 — Number of isolates/percentage per year and subtype. (DOCX) [file pone.0016902.s002.docx]

| Year | CRF01_AE | Subtype B | CRF15_AE |
| --- | --- | --- | --- |
| 1999 (5) | 5/100 | 0 | 0 |
| 2000 (73) | 63/86.3 | 9/12.3 | 1/1.4 |
| 2001 (58) | 46/79.3 | 10/17.2 | 2/3.5 |
| 2002 (69) | 59/85.5 | 9/13.0 | 1/1.5 |
| 2003 (10) | 9/90.0 | 1/10 | 0 |
| Total (215) | 182/84.7 | 29/13.4 | 4/1.9 |
