# Supplementary material for: Phylodynamics of HIV-1 from a Phase III AIDS Vaccine Trial in Bangkok, Thailand
Source: PLoS One. 2011 Mar 10;6(3):e16902. doi: 10.1371/journal.pone.0016902 (PMC3053363; doi:10.1371/journal.pone.0016902)
Supplement: Table S2 — Phylogenetic transmission clusters. Estimated date of infection and clinical site for subtype B and CRF01_AE. (DOCX) [file pone.0016902.s003.docx]

| Subtype B | | | |
| --- | --- | --- | --- |
| Cluster | Isolate | Date | Clinic |
| 1 | 32 | 2001.6.18 | 104 |
| 1 | 101 | 2001.10.17 | 109 |
| 1 | 104 | 2001.8.2 | 107 |
| 1 | 140 | 2001.6.25 | 115 |
| 1 | 186 | 2000.10.12 | 117 |
| 2 | 45 | 2002.3.28 | 105 |
| 2 | 47 | 2001.11.1 | 105 |
| 2 | 50 | 2002.1.8 | 105 |
| 3 | 60 | 2000.3.21 | 106 |
| 3 | 145 | 2001.8.3 | 114 |
| CRF01_AE | | | |
| Cluster | Isolate | Date | Clinic |
| 1 | 2 | 2002.2.11 | 101 |
| 1 | 128 | 2000.12.21 | 110 |
| 1 | 132 | 2001.3.8 | 110 |
| 1 | 158 | 2001.12.27 | 110 |
| 1 | 172 | 2002.3.26 | 110 |
| 2 | 7 | 2001.11.27 | 101 |
| 2 | 110 | 2000.3.27 | 115 |
| 3 | 12 | 2002.2.20 | 102 |
| 3 | 42 | 2001.2.28 | 104 |
| 3 | 86 | 2001.3.23 | 108 |
| 3 | 108 | 2000.1.11 | 116 |
| 3 | 170 | 2002.3.14 | 110 |
| 3 | 188 | 2001.8.24 | 117 |
| 4 | 9 | 2000.1.27 | 102 |
| 4 | 189 | 2002.9.20 | 103 |
| 5 | 13 | 2000.9.11 | 102 |
| 5 | 129 | 2001.2.5 | 116 |
| 6 | 14 | 2000.10.11 | 102 |
| 6 | 121 | 2000.8.18 | 116 |
| 6 | 134 | 2001.3.20 | 116 |
| 7 | 16 | 1999.9.17 | 103 |
| 7 | 17 | 2000.2.25 | 103 |
| 8 | 19 | 2000.3.3 | 103 |
| 8 | 20 | 2000.4.24 | 103 |
| 9 | 21 | 2002.8.30 | 101 |
| 9 | 161 | 2002.11.8 | 115 |
| 10 | 23 | 2002.8.7 | 102 |
| 10 | 106 | 1999.11.16 | 114 |
| 11 | 24 | 2002.8.7 | 102 |
| 11 | 155 | 2001.11.21 | 115 |
| 11 | 174 | 2002.5.14 | 114 |
| 11 | 178 | 2002.6.11 | 114 |
| 12 | 25 | 2002.6.17 | 103 |
| 12 | 68 | 2002.6.5 | 106 |
| 12 | 113 | 2000.6.14 | 114 |
| 12 | 116 | 2000.7.14 | 114 |
| 12 | 146 | 2001.8.17 | 114 |
| 12 | 182 | 2002.8.28 | 114 |
| 12 | 198 | 2002.11.19 | 106 |
| 13 | 27 | 2002.2.15 | 103 |
| 13 | 29 | 2002.4.10 | 103 |
| 13 | 97 | 2000.3.9 | 109 |
| 13 | 118 | 2000.8.2 | 110 |
| 13 | 125 | 2000.8.22 | 110 |
| 14 | 34 | 2000.1.12 | 104 |
| 14 | 37 | 2000.3.29 | 104 |
| 15 | 49 | 2002.2.27 | 105 |
| 15 | 175 | 2002.5.17 | 111 |
| 16 | 51 | 2002.7.8 | 105 |
| 16 | 208 | 2003.1.9 | 105 |
| 16 | 209 | 2003.2.27 | 117 |
| 17 | 56 | 2000.6.13 | 106 |
| 17 | 61 | 2000.10.2 | 106 |
| 18 | 26 | 2001.8.3 | 103 |
| 18 | 65 | 2000.12.22 | 106 |
| 18 | 70 | 2000.1.26 | 106 |
| 19 | 73 | 2000.8.25 | 106 |
| 19 | 139 | 2000.10.30 | 114 |
| 20 | 75 | 2001.8.23 | 107 |
| 20 | 171 | 2002.3.21 | 112 |
| 21 | 78 | 2000.11.28 | 107 |
| 21 | 165 | 2002.2.5 | 115 |
| 21 | 192 | 2002.10.4 | 113 |
| 22 | 79 | 2002.5.23 | 107 |
| 22 | 176 | 2002.6.4 | 115 |
| 22 | 183 | 2002.9.5 | 111 |
| 23 | 80 | 2001.4.12 | 107 |
| 23 | 143 | 2001.7.17 | 116 |
| 24 | 83 | 2000.9.22 | 107 |
| 24 | 164 | 2002.2.1 | 111 |
| 25 | 84 | 2001.3.15 | 107 |
| 25 | 85 | 2001.2.22 | 107 |
| 25 | 117 | 2000.7.26 | 109 |
| 25 | 168 | 2002.2.25 | 113 |
| 26 | 58 | 2002.1.2 | 106 |
| 26 | 89 | 2000.3.8 | 108 |
| 26 | 90 | 2000.3.13 | 108 |
| 27 | 96 | 2001.12.3 | 109 |
| 27 | 103 | 2001.11.26 | 109 |
| 28 | 112 | 2000.6.12 | 115 |
| 28 | 214 | 2003.4.17 | 111 |
| 29 | 135 | 2001.4.10 | 113 |
| 29 | 181 | 2002.8.15 | 114 |
| 30 | 39 | 2000.2.17 | 104 |
| 30 | 64 | 2000.11.28 | 106 |
| 30 | 147 | 2001.8.27 | 110 |
| 30 | 151 | 2001.10.18 | 110 |
| 30 | 202 | 2002.1.24 | 104 |
| 31 | 153 | 2001.11.7 | 115 |
| 31 | 173 | 2002.4.9 | 115 |
| 32 | 205 | 2002.12.24 | 103 |
| 32 | 216 | 2003.5.20 | 107 |
